# VX-770, C_act_-A1, and Increased Intracellular cAMP Have Distinct Acute Impacts upon CFTR Activity

**DOI:** 10.3390/ijms26020471

**Published:** 2025-01-08

**Authors:** Heidi J. Nick, Sarah E. Christeson, Preston E. Bratcher

**Affiliations:** 1 Department of Pediatrics, National Jewish Health, Denver, CO 80206, USA; nickh@njhealth.org (H.J.N.); christesons@njhealth.org (S.E.C.); 2 Department of Pediatrics, University of Colorado Anschutz Medical Campus, Aurora, CO 80045, USA

**Keywords:** cystic fibrosis, CFTR, VX-770, ivacaftor, C_act_-A1, cAMP, CFTR modulator, airway ion transport

## Abstract

The cystic fibrosis transmembrane conductance regulator (CFTR) is an anion channel that is dysfunctional in individuals with cystic fibrosis (CF). The permeability of CFTR can be experimentally manipulated though different mechanisms, including activation via inducing the phosphorylation of residues in the regulatory domain as well as altering the gating/open probability of the channel. Phosphorylation/activation of the channel is achieved by exposure to compounds that increase intracellular cAMP, with forskolin and IBMX commonly used for this purpose. C_act_-A1 is a unique CFTR activator that does not increase intracellular cAMP, and VX-770 (ivacaftor) is a CFTR potentiator that is used experimentally and therapeutically to increase the open probability of the channel. Using primary human nasal epithelial cell (HNEC) cultures and Fischer rat thyroid (FRT) epithelial cells exogenously expressing functional CFTR, we examined the impact of VX-770, C_act_-A1, and forskolin/IBMX on CFTR activity during analysis in an Ussing chamber. Relative contributions of these compounds to maximal CFTR activity were dependent on order of exposure, the presence of chemical and electrical gradients, the level of constitutive CFTR function, and the cell model tested. Increasing intracellular cAMP appeared to change cellular functions outside of CFTR activity that resulted in alterations in the drive for chloride through CFTR. These results demonstrate that one can utilize combinations of small-molecule CFTR activators and potentiators to provide detailed characterization of CFTR-mediated ion transport in primary HNECs and properties of these modulators in both primary HNECs and FRT cells. Future studies using these approaches may assist in the identification of novel defects in CFTR function and the identification of modulators with unique impacts on CFTR-mediated ion transport.

## 1. Introduction

Cystic fibrosis (CF) is a rare, inherited disease with clinical manifestations in multiple organs, including the lung, pancreas, intestine, kidneys, and liver [1]. CF is an autosomal recessive disease that is exhibited when both alleles of the cystic fibrosis transmembrane conductance regulator (*CFTR*) harbor mutations that result in improperly functioning and/or expressed proteins. CFTR functions as an anion channel that is expressed in epithelial cells, and CF-causing mutations in the channel cause a defect in transepithelial chloride transport. In the lung, the outcome of impaired CFTR function in the airway epithelium is dehydration of mucus, resulting in airway obstruction and debilitating respiratory infections; the overall consequence of these presentations is a decrease in lung function.

CFTR modulators are a novel class of small-molecule therapeutics that directly address the cause of CF by increasing the function and/or expression of CFTR [2,3]. These molecules have been subclassified based on the specific CFTR defect that they address: correctors are molecules with the ability to increase plasma membrane expression of the F508del mutant CFTR, while potentiators are defined as molecules that increase the open probability of the channel and are used to increase chloride conductance through mutant CFTRs that are defective in channel gating. VX-770 is a highly efficacious potentiator that is used clinically to treat people with CF who have gating and residual function CFTR mutations, including the more common gating mutation, G551D [4,5,6,7,8,9,10,11]. Additionally, as the most common CF-causing CFTR mutation, F508del, also has a channel gating defect, VX-770 is included in combination therapeutics containing corrector molecules [12,13,14,15,16]. Currently, all FDA-approved CFTR modulators are composed of or include VX-770.

The permeability of CFTR is tightly controlled through a regulatory (R) domain in which several residues require phosphorylation to induce a conformational change that opens the channel pore. This phosphorylation and subsequent opening are referred to as “activation”. “Gating” of ion movement through the channel is independent of “activation” and occurs through ATP binding to the nucleotide binding domains (NBDs), resulting in dimerization and another confirmational change [17]. Thus, maximal ion conductance through CFTR requires both opening of the NBD “gate” (i.e., “potentiation”) and phosphorylation of the channel (i.e., “activation”). Phosphorylation of CFTR is mediated by protein kinase A (PKA), and, therefore, inducing the phosphorylation of CFTR can be easily achieved experimentally by increasing intracellular cAMP levels, thereby activating PKA. Forskolin and 3-isobutyl-1-methylxanthine (IBMX) are two compounds commonly utilized in CF research to activate CFTR through raising intracellular cAMP by activating adenylate cyclase and inhibiting phosphodiesterase, respectively. While some CF-causing CFTR mutants require a potentiator such as VX-770 in order to reach maximal ion conductance, completely functional CFTR is able to reach this maximum after cells are exposed to saturating concentrations of forskolin and/or IBMX.

Several small molecules have been identified that activate CFTR [18,19,20,21]. One of these molecules, C_act_-A1, has the ability to activate functional CFTR through a mechanism that is independent of increasing intracellular cAMP [22]. C_act_-A1 also activates both F508del and G551D CFTR mutants and is able to increase CFTR-mediated currents above that induced by increasing intracellular cAMP, indicating that it has potentiator activity as well. The potentiator activity of VX-770 is due to its direct binding to CFTR and stabilizing the open confirmational state present before ATP hydrolysis [23,24]. While the mechanisms of the C_act_-A1-mediated impacts on CFTR activity have not been fully described, it has been demonstrated that exposure to C_act_-A1 and VX-770 produces distinct effects on the chloride conductance through CFTR [22]. Importantly, VX-770 alone induces weak responses in F508del CFTR, while C_act_-A1 stimulates CFTR-mediated currents similarly but distinctly to that induced by the cAMP-induced activation of CFTR. Independently, both VX-770 and C_act_-A1 induce weak responses in G551D CFTR, but when combined, their impacts are highly synergistic.

VX-770, C_act_-A1, and forskolin/IBMX increase CFTR-mediated ion transport through distinct mechanisms and, as such, responses to these compounds provide unique readouts of CFTR function. The aim of this study was to characterize the functional impacts of these compounds when applied to epithelial cell cultures in varying sequential orders and in the presence of diverse electrochemical gradients to examine their potential informativeness during analysis of CFTR function. Increasing the number of valuable readouts of CFTR function could be beneficial for examining outcomes of CFTR modulator treatments and for defining uncharacterized functional defects present in mutant CFTRs.

## 2. Results

### 2.1. Magnitude of Responses to VX-770 and C_act_-A1 Are Dependent on Order of Exposure, Strength of Electrical and Chemical Gradients, and Constitutive CFTR Activity Levels in HNECs

**Figure 1:**
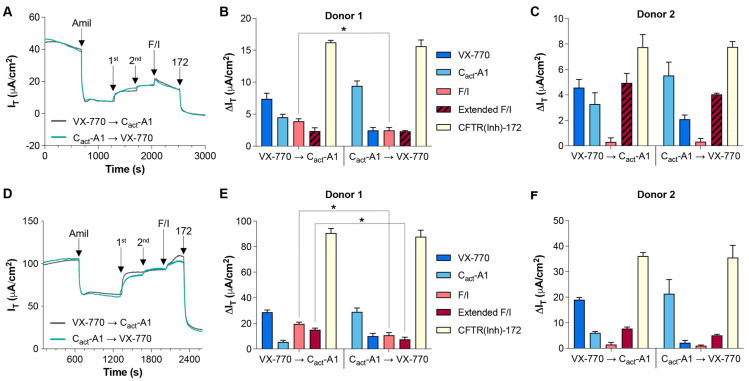
VX-770 and C_act_-A1 induce similar but distinct responses in HNECs. Transepithelial currents (I_T_) in HNECs from healthy donors were analyzed in an Ussing chamber in the absence (**A**) or presence (**D**) of a chloride gradient with exposure to either VX-770 followed by C_act_-A1 or C_act_-A1 followed by VX-770 (representative tracings obtained from “Donor 1” cultures are shown). Changes in transepithelial current (∆I_T_) values in response to amiloride (“Amil”), VX-770/C_act_-A1 (“1st” or “2nd”, depending on the order listed in the panel legends), forskolin/IBMX (“F/I”), and CFTR(inh)-172 (“172”) are presented for HNECs generated from two donors and analyzed in the absence (**B**,**C**) or presence (**E**,**F**) of a chloride gradient. All values are maximal responses except for “Extended F/I” values, which were calculated using the value obtained immediately before the exposure to CFTR(inh)-172. All values are absolute values, and “Extended F/I” values with striped bars indicate that the change in current was negative. * *p* < 0.05. *n* = 4 technical replicates from a single donor per group.

In order to further characterize the cellular responses to the small-molecule CFTR modulators VX-770 and C_act_-A1, human nasal epithelial cells (HNECs) were exposed sequentially to the compounds under various conditions, including under symmetrical chloride and in the presence of an apical chloride gradient (Figure 1), which is known to impact CFTR-mediated chloride conductance [25,26]. Regardless of the order of exposure to VX-770 and C_act_-A1 and the conditions for the analysis, the response to the first compound was greater than the response to the second, although the relative magnitudes of the responses did vary depending on order, condition, and donor; under symmetrical chloride, the response to VX-770 was not significantly different from the response to subsequent C_act_-A1 for Donor 2 (Figure 1C). As the magnitude of the responses to both compounds decreased when utilized second as compared to first, the functional impact on CFTR-mediated transepithelial current (I_T_) overlapped between the two compounds. However, responses to the compounds clearly indicated that their mechanisms of action are unique. With symmetrical chloride, VX-770 and C_act_-A1 induced responses that were similar in magnitude when utilized in the order of VX-770 then C_act_-A1, but, when added in reverse, the impact of C_act_-A1 was much more dominant than the subsequent response to VX-770. Importantly, while HNECs analyzed under symmetrical chloride responded to forskolin/IBMX (F/I) with a transient increase in current, the overall response to the post-VX-770/C_act_-A1 exposure to F/I was a decrease in current (Figure 1B,C, striped bars). However, under an apical chloride gradient, all cultures responded to F/I with an increase in current. Interestingly, in HNECs generated from Donor 1, peak responses to F/I were significantly greater when VX-770 exposure occurred before C_act_-A1 under both symmetrical and gradient conditions, and the extended response to F/I was greater under gradient conditions. In all cases, the responses to the CFTR inhibitor CFTR(inh)-172 were not significantly different when the order of VX-770 and C_act_-A1 exposures was alternated.

**Figure 2:**
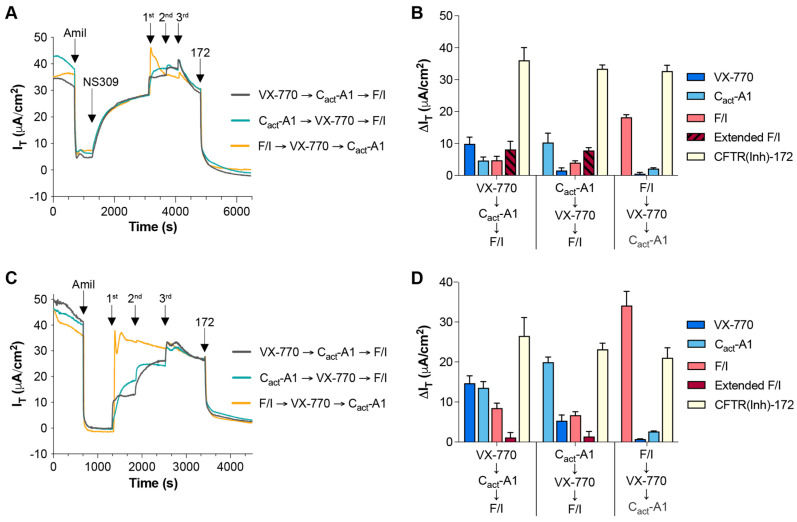
Increasing the electrical drive for CFTR-mediated chloride transport and decreasing constitutive CFTR activity in HNECs distinctly altered the responses to VX-770 and C_act_-A1. Transepithelial currents (I_T_) in HNECs from a healthy donor (cultures were technical replicates of the Donor 1 cultures utilized for Figure 1) were analyzed in an Ussing chamber with acute exposure to NS309 (**A**) or after 24 h of exposure to forskolin (**C**). Changes in transepithelial current (∆I_T_) values in response to the listed compound are presented for cultures exposed to acute NS309 (**B**) or 24 h of forskolin (**D**). All values are maximal responses except for “Extended F/I” values, which were calculated using the value obtained immediately before the exposure to CFTR(inh)-172. All values are absolute values, and “Extended F/I” values with striped bars indicate that the change in current was negative. *n* = 4 technical replicates from a single donor per group.

In addition to comparisons made in the presence of a strong chemical drive for chloride transport through CFTR, NS309 was utilized to stimulate small/intermediate-conductance calcium-activated potassium channels and provide a strong electrical drive for chloride transport through CFTR [27]. Responses to sequential VX-770 and C_act_-A1 mirrored those observed in the absence of NS309 (Figure 2A,B), and the response to F/I post-VX-770/C_act_-A1 ended in an overall decrease in I_T_. In order to examine whether levels of constitutively active CFTR have a significant influence on the responses to VX-770 and C_act_-A1, a previously reported approach of treating cultures with forskolin for 24 h prior to analysis was utilized to decrease the amount of constitutively active CFTR [28,29]. HNECs treated with forskolin before analysis had larger current responses to VX-770 and C_act_-A1 than those analyzed without forskolin, as shown in Figure 1B (Figure 2C,D), particularly with VX-770 when applied as the initial stimulus. Similarly to HNECs analyzed under an apical chloride gradient, the “Extended F/I” current response values were positive increases in current in HNECs pretreated with forskolin. Notably, the total CFTR activity (as measured by responses to CFTR(inh)-172) in HNECs treated with forskolin for 24 h prior to analysis was greater than in matched HNECs without this treatment (Figure 1B, Donor 1); the increase in CFTR-mediated currents may have been due to increased expression of CFTR on the plasma membrane due to stimulated exocytosis [30,31], and this may have also impacted the differences observed in the responses to VX-770 and C_act_-A1.

**Figure 3:**
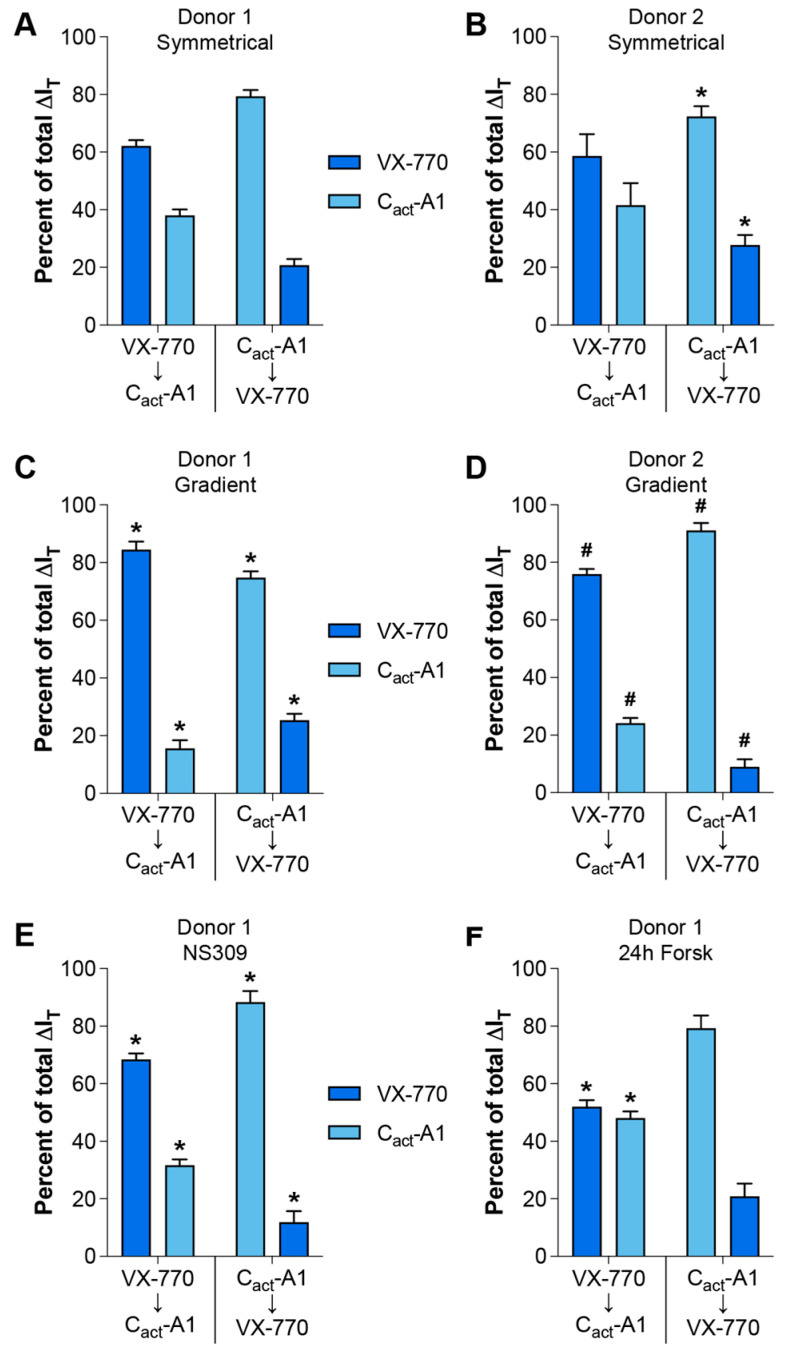
Conditions for assessing the responses to VX-770 and C_act_-A1 significantly altered their relative impacts on CFTR-mediated currents. Changes in transepithelial currents (ΔI_T_) in response to VX-770 and C_act_-A1 are expressed as a percentage of the total response to both compounds. Values were calculated from the data presented in Figure 1 (**A**–**D**) and Figure 2 (**E**,**F**). * *p* < 0.05 as compared to the same values for Donor 1 cultures assayed under symmetrical chloride (**A**). ^#^ *p* < 0.05 as compared to the same values for Donor 2 cultures assayed under symmetrical chloride (**B**). *n* = 4 technical replicates from a single donor per group.

In order to simplify comparisons amongst the conditions under which VX-770 and C_act_-A1 responses were analyzed, changes in values in response to drugs were normalized to the change in value as measured in cultures generated from Donor 1 analyzed under symmetrical conditions (Figure 3); the exceptions to this are the values obtained using cultures generated from Donor 2 analyzed under gradient conditions, for which the values were normalized to Donor 2 cultures analyzed under symmetrical conditions. In all cases, the response to the first drug was larger than the response to the second drug, with the single exception of VX-770 followed by C_act_-A1 in the cultures pretreated for 24 h with forskolin, for which the responses were not significantly different, although the difference was approaching statistical significance (*p* = 0.0524). When compared to the values from Donor 1 cultures analyzed under symmetrical chloride, the percentages of the total I_T_ response attributable to VX-770 and C_act_-A1 in the Donor 1 cultures analyzed under a chloride gradient, with NS309 treatment, and with 24 h forskolin pretreatment were significantly altered, with the exception of the responses to C_act_-A1 then VX-770 in the cultures pretreated with forskolin. This demonstrates that the presence of a chloride gradient, the activity of other ion channels, and the levels of constitutive CFTR activity in primary HNEC cultures can significantly change the relative impacts of VX-770 and C_act_-A1, and that these impacts can be sequence-dependent. When comparing cultures generated from Donor 2 analyzed under symmetrical chloride (Figure 3B) with those from Donor 1 (Figure 3A), the relative contributions of VX-770 and C_act_-A1 to the total I_T_ change were not different when the VX-770 exposure occurred prior to C_act_-A1, but the relative contributions were significantly different when C_act_-A1 was the initial treatment. Similarly to what was observed in cultures generated from Donor 1, the presence of a chloride gradient significantly impacted the relative contributions of VX-770 and C_act_-A1 to the total I_T_ change in cultures generated from Donor 2 (Figure 3D). These results demonstrate that there are culture-to-culture variations in the responses to VX-770 and C_act_-A1 that are dependent on the order of exposure and the absence or presence of a chloride gradient.

### 2.2. VX-770 and C_act_-A1 Differentially Impact I_T_ and Transepithelial Potential Difference in FRT Cells Expressing Functional CFTR

**Figure 4:**
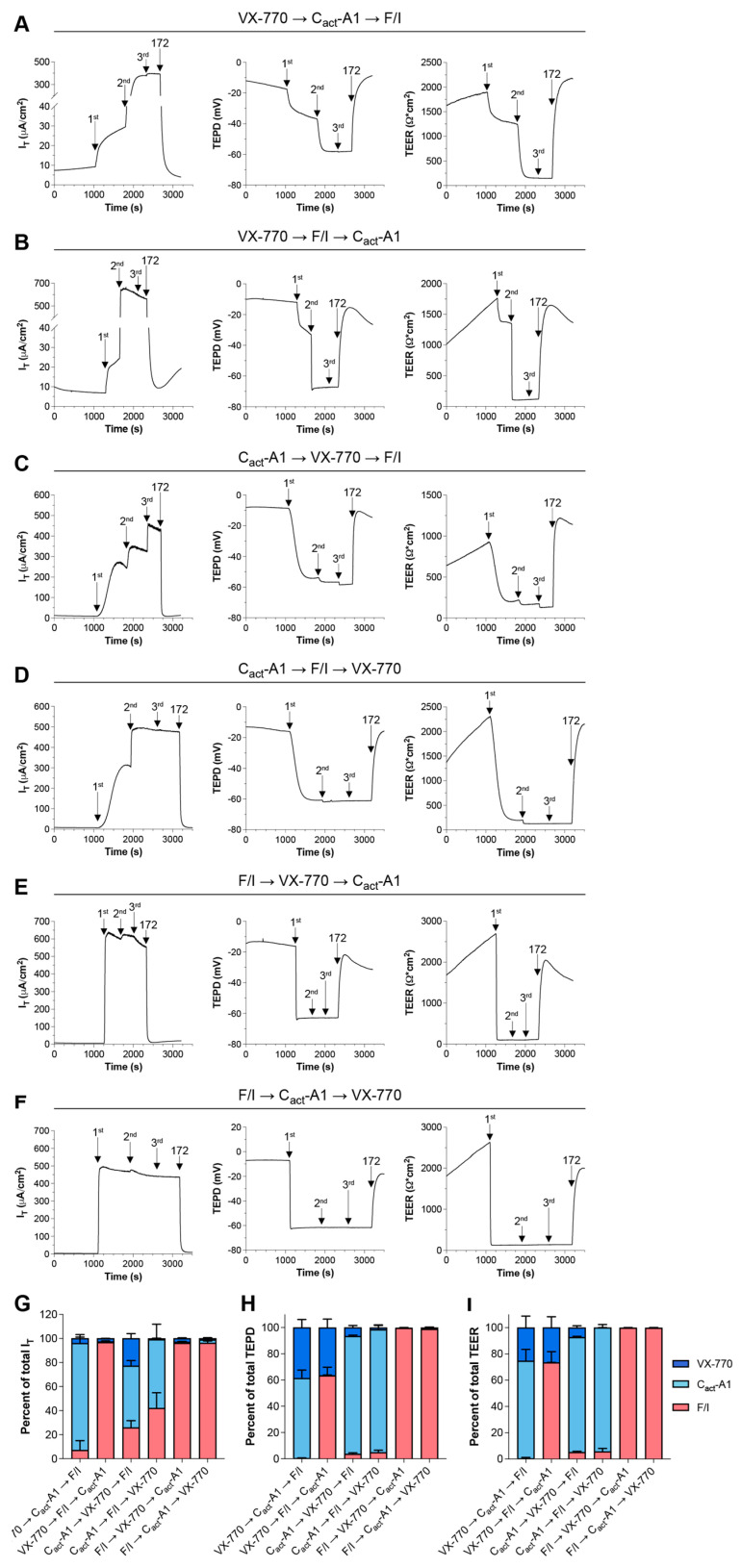
The magnitude of the responses to C_act_-A1 were larger than the responses to VX-770 in FRT cells expressing functional CFTR. FRT cultures were analyzed in an Ussing chamber in the presence of a chloride gradient with exposures to VX-770, C_act_-A1, and F/I in various sequences (**A**–**F**). (**G**–**I**) Changes in values in response to VX-770, C_act_-A1, and F/I are expressed as percentages of the total response to all three compounds. *n* = 4 technical replicates per group.

To further examine the functional impacts of VX-770 and C_act_-A1 on CFTR, responses to these compounds were analyzed in Fischer rat thyroid (FRT) cells exogenously expressing functional CFTR. In this model, the results more clearly demonstrate changes in CFTR activity, as CFTR is expressed at a very high level and there is very little constitutive CFTR activity in these cells. Other potential differences in CFTR function may exist between FRT cells and HNECs, and these may also contribute to model-specific variations in responses, as has been previously described ([32], reviewed in [2,33]). When applied as the first treatment, VX-770-induced changes in I_T_ were minimal, while larger changes were observed in both transepithelial potential difference (TEPD) and transepithelial electrical resistance (TEER) (Figure 4A,B,G–I). This suggests that VX-770 increased the opening of CFTR, but did not induce active transport of ions through the channel. C_act_-A1-induced changes in I_T_ were largest when preceded immediately by VX-770 (Figure 4A,C,G), but cultures reached near-maximal hyperpolarization values after C_act_-A1 exposure regardless of whether or not treatment followed VX-770 (Figure 4A,C,D,H). As TEER values reached a minimum after C_act_-A1, this suggests that C_act_-A1 fully opened and activated the CFTR channel. When F/I was applied first, there were no significant responses to subsequent C_act_-A1 or VX-770 exposures, similarly to what was observed in HNECs (Figure 4E–I). Interestingly, the order of treatment impacted the results, as VX-770 then C_act_-A1 was able to stimulate maximal CFTR-mediated ion transport (as demonstrated by a lack of a further increase when F/I was applied), but this was not observed for C_act_-A1 followed by VX-770. Also of interest is the observation that F/I caused an increase in I_T_ after C_act_-A1, but did not impact the TEPD or TEER; this suggests that F/I altered the function of other cellular targets that increased the flow of chloride through CFTR.

### 2.3. Pretreatment with 10 nM Forskolin Increases the I_T_ Responses to VX-770 and C_act_-A1 in FRT Cells Expressing Functional CFTR

**Figure 5:**
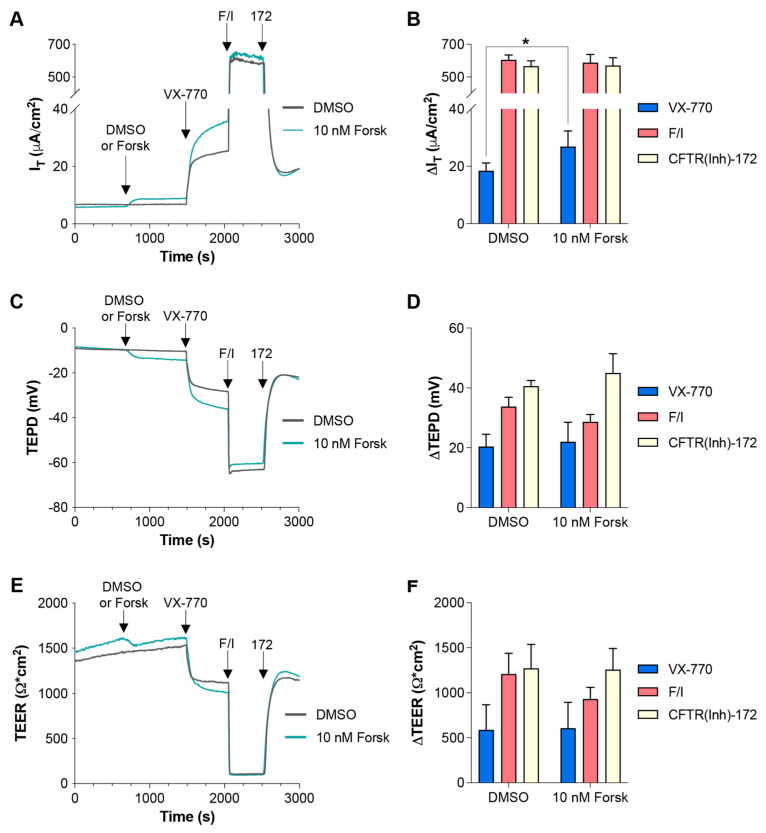
Increasing intracellular cAMP enhanced I_T_ changes in responses to subsequent VX-770 exposure in FRT cells expressing functional CFTR. (**A**,**C**,**E**) FRT cultures were analyzed in an Ussing chamber with sequential exposure to either DMSO (gray line) or 10 nM forskolin (teal line), followed by VX-770, F/I, and CFTR(inh)-172. (**B**,**D**,**F**) Changes in values in response to the listed compound are presented. * *p* < 0.05. *n* = 6 technical replicates per group.

**Figure 6:**
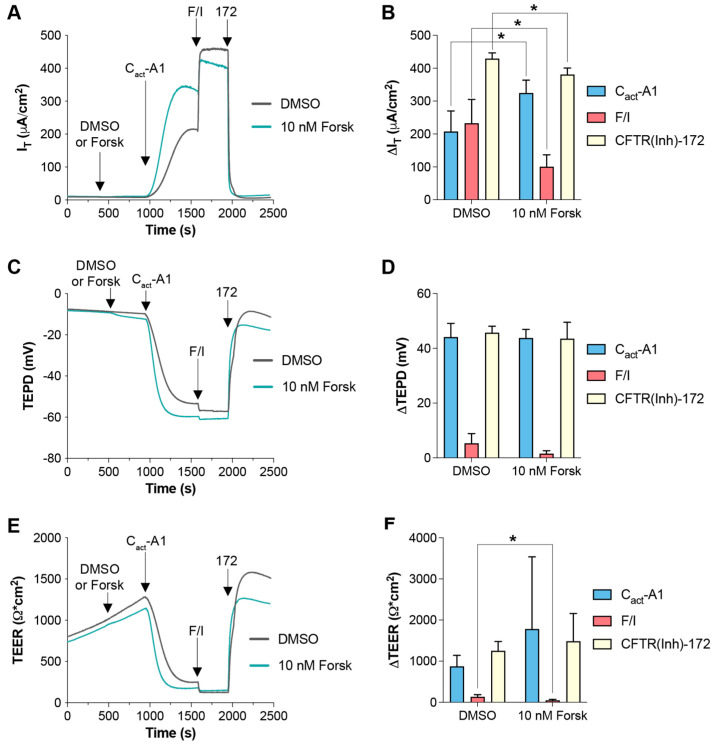
Increasing intracellular cAMP enhanced I_T_ changes in responses to subsequent C_act_-A1 exposure in FRT cells expressing functional CFTR. (**A**,**C**,**E**) FRT cultures were analyzed in an Ussing chamber with sequential exposure to either DMSO (gray line) or 10 nM forskolin (teal line), followed by C_act_-A1, F/I, and CFTR(inh)-172. (**B**,**D**,**F**) Changes in values in response to the listed compound are presented. * *p* < 0.05. *n* = 4 technical replicates per group.

In the data presented in Figure 4, it appears that C_act_-A1 fully activated/opened the CFTR channels in FRT cells, as demonstrated by the minimization of TEER that was not further lowered upon exposure to F/I. However, F/I exposure increased I_T_ when applied after C_act_-A1. To investigate whether raising intracellular cAMP levels was resulting in cellular changes via targets other than CFTR, FRT cells were exposed to a low concentration of forskolin to induce slight increases in intracellular cAMP, and they were then treated with either VX-770 (Figure 5) or C_act_-A1 (Figure 6) [34]. For both VX-770 and C_act_-A1, pretreatment with 10 nM forskolin increased the magnitude of the change in I_T_ upon exposure to the subsequent drug, but had no significant impact on the changes in TEPD and TEER. As VX-770 and C_act_-A1 had no impact on cells expressing normal CFTR that were exposed to saturating levels of F/I, these results suggest that the increase in CFTR-mediated I_T_ (and lack of increase in TEPD and TEER) observed in the presence of 10 nM forskolin was the result of the cAMP-induced modulation of non-CFTR targets, which increased the chemical and/or electrical drive for chloride through CFTR.

### 2.4. Response to VX-770, C_act_-A1, and F/I Are Variably Decreased in the Presence of CFTR(inh)-172 and in FRT Cells with No Exogenous CFTR Expression

**Figure 7:**
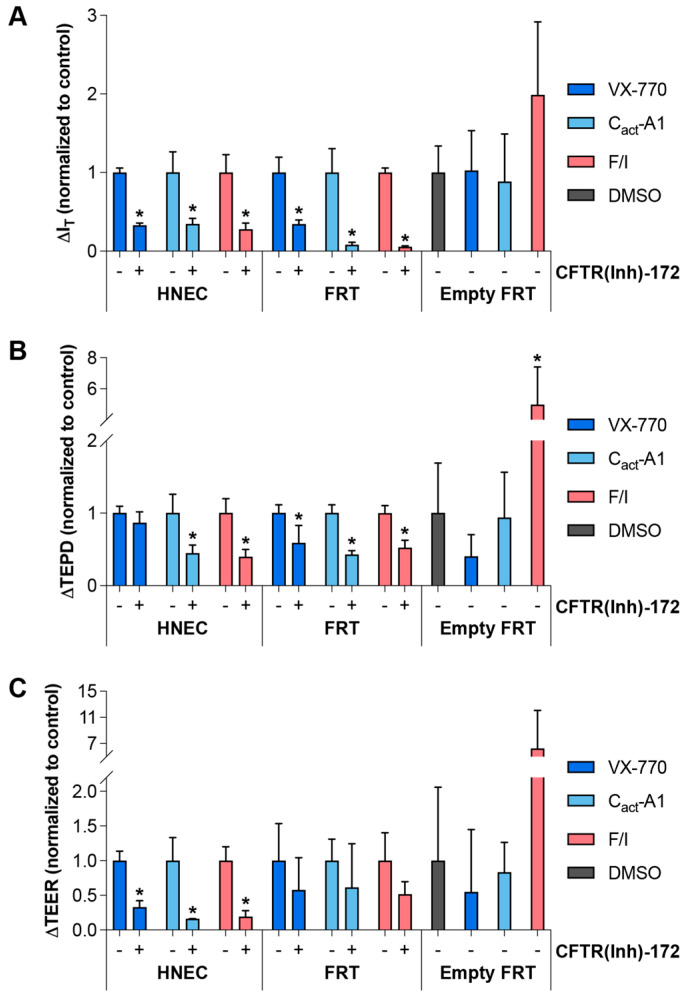
Inhibition or absence of CFTR decreased the responses to VX-770, C_act_-A1, and F/I to varying degrees. (**A**–**C**) HNECs from a healthy donor (cultures were technical replicates of the Donor 2 cultures utilized for Figure 1), FRT cells expressing functional CFTR (“FRT”), and FRT cells stably transfected with an empty vector (“Empty FRT”) were analyzed in an Ussing chamber in the presence of a chloride gradient with acute exposure to VX-770, C_act_-A1, or F/I in the absence (indicated with a “−” below the columns) or presence (indicated with a “+”) of CFTR(inh)-172. Changes in values in response to treatments for HNECs and FRT cells were normalized to the changes in values in the absence of CFTR(inh)-172. Changes in values in response to treatments for Empty FRT were normalized to changes in values in response to DMSO alone. * *p* < 0.05. *n* = 3–5 technical replicates from a single donor per group.

To further examine whether non-CFTR targets would be impacted by VX-770, C_act_-A1, and F/I, responses to these molecules were measured in the presence of CFTR(inh)-172. In all cases, CFTR(inh)-172 significantly reduced the drug responses in I_T_ and TEPD, with the exception of the TEPD response to VX-770 in HNECs (Figure 7). The degree of inhibition was less than expected for VX-770 and C_act_-A1, given that both molecules are believed to interact directly with the CFTR protein. This may have been due to incomplete inhibition of CFTR by 20 μM CFTR(inh)-172. Alternatively, it has been suggested that VX-770 and C_act_-A1 may compete with CFTR(inh)-172 for binding [22,35], and this would provide an explanation for the observed results. In order to address this potential fault with the experimental approach, the responses to drugs were tested in FRT cells transfected with an empty vector (i.e., these cells did not express exogenous CFTR). In this model, only F/I induced a response that was significantly different than the response to the DMSO alone, and this was only observed in the TEPD (Figure 7). Furthermore, the response to CFTR(inh)-172 after the various stimuli in these FRT cells was also only significantly different from controls in the F/I-exposed cultures (Figure S4), indicating that there may have been endogenously-expressed CFTR present in these, that the CFTR present in these cells was constitutively active, and that F/I increased the drive for chloride through the constitutively active CFTR. These observations provide additional evidence that suggests that F/I impacts targets other than CFTR.

## 3. Discussion

In both primary HNECs and the FRT cell model, VX-770, C_act_-A1, and F/I induced distinct electrophysiological responses. The relative contributions they each had to the induction of CFTR-mediated currents was dependent on multiple factors, including order of exposure, the presence and magnitude of electrical and chemical gradients, and the level of constitutive CFTR activity in the culture. It is possible that each of these factors may have impacted the structural conformation of CFTR and thereby altered the interactions of VX-770 and C_act_-A1 with CFTR. Differences in the response to VX-770 and C_act_-A1 may have been due to each having a unique binding site within the CFTR channel (potentially in the same protein domain), and they may have competed allosterically with each other as well as CFTR(inh)-172 for binding [22,35]. Future studies will further explore the mechanisms behind our observations.

F/I alone is capable of maximizing CFTR-mediated currents in cells with fully functional CFTR, but its impact on currents after exposure to VX-770 and C_act_-A1 dramatically depends on the conditions for analysis. When HNECs are bathed in symmetrical chloride buffers, post-VX-770/C_act_-A1 exposure to F/I results in an overall decrease in current, and this occurs in the presence and absence of an additional electrical gradient provided by potassium channel activation using NS309. However, in the presence of a chemical chloride gradient or when constitutive CFTR activity has been decreased using 24 h pre-exposure to forskolin, F/I treatment following VX-770/C_act_-A1 results in an increase in current. This suggests that F/I is affecting targets other than CFTR, and those targets are differentially impacted by electrical/chemical driving forces and raised intracellular cAMP for 24 h before analysis. This would explain how F/I could impact targets in a manner that results in an overall increase in the drive for chloride through CFTR or, under different circumstances, an overall decrease in the drive for chloride through CFTR. Furthermore, the relative magnitudes of the “Extended F/I” values were highly variable from donor to donor (Figure 1), and it is probable that the expression/function of non-CFTR targets impacted by raised intracellular cAMP are variable from culture to culture. If this is the mechanism responsible, donor-to-donor variability in measures of CFTR activity may be decreased if only VX-770 and C_act_-A1 are utilized to stimulate CFTR-mediated currents in primary airway epithelial cells. F/I or similar stimuli that increase intracellular cAMP may be excluded or utilized after VX-770/C_act_-A1 to monitor the expression/function of non-CFTR targets.

Importantly, the changes in I_T_ and TEER/TEPD in response to VX-770 and C_act_-A1 were not complementary in FRT cells expressing functional CFTR. As shown in Figure 4, an initial exposure to VX-770 resulted in an extremely small increase in current (4.00 ± 1.07% of total current induced), but a much larger decrease in TEER (25.4 ± 8.75% of total decrease in TEER) and a change in TEPD (38.7 ± 6.20% of total TEPD change). If the drive for chloride currents is dramatically increased by raised intracellular cAMP in a CFTR-independent manner, this may explain how VX-770 exposure changed the CFTR-dependent TEPD to a large degree without inducing a large change in current.

Two experiments described herein mirror experiments performed in previous reports, with different observations made in the current study. In the initial report describing the discovery and characterization of C_act_-A1 [22], stimulation of FRT cells expressing function CFTR with 10 μM C_act_-A1 resulted in currents very close to the maximal levels observed, with only a slight increase upon the subsequent exposure to 30 μM C_act_-A1 and, importantly, 20 μM forskolin. In a comparable experiment described in this study (Figure 4D,G), exposure to 10 μM C_act_-A1 increased currents to 57.2 ± 12.8% of maximum, with subsequent F/I increasing the current by a further 42.0 ± 12.9%. Variations in approaches may have resulted in these differences, including the use of IBMX in the present study and analysis of cells at room temperature and subjecting cells to a 0 mV voltage clamp in the previous study. Interestingly, in the previous study, the basolateral membrane was permeabilized prior to stimulation, eliminating intracellular chloride as a limiting substrate and potential chemical driver in those experiments. If intracellular cAMP is impacting the chemical drive for chloride in the presence of an intact basolateral membrane, this would explain the observed results.

In a separate experiment performed similarly in the current study to a previous report [36], FRT cells expressing functional CFTR were exposed to 10 nM forskolin prior to stimulation with 1 μM VX-770. In the prior study, this sequence resulted in a near-maximal induction of currents, while in the present study (Figure 5A,B), only 4.35 ± 0.629% of the maximum current was reached with this sequence. The major difference in approach between these two experiments was the use of short-circuit conditions (0 mV voltage clamp) in the previous report and open-circuit conditions (0 μA current clamp) in this report. Future experiments may reveal whether the impact of VX-770 on CFTR is dependent on TEPD. For both previous publications mentioned here [22,36], observations from experiments with low concentrations of forskolin prior to stimulation with VX-770 [36] or C_act_-A1 [22] were interpreted as demonstrations that phosphorylation levels of CFTR alter the responses to these compounds. Herein, we interpret these results as demonstrations that increasing intracellular cAMP increases the drive for chloride through CFTR, thereby increasing the magnitude of the response to compounds that modulate CFTR function. This could occur through the activation of potassium channels, for example (reviewed in [37]). Furthermore, we suggest that the results of the experiment in which HNECs were exposed to forskolin for 24 h prior to analysis (resulting in a decrease in constitutive CFTR activity and, presumably, levels of phosphorylated CFTR) indicate that, for fully functional CFTR, the impacts of VX-770 and C_act_-A1 on CFTR-mediated ion transport are independent of phosphorylation.

Although the experiments conducted in the present study were performed in the context of properly functioning CFTR, the approach of utilizing the sequential additions of VX-770 and C_act_-A1 during the analysis of mutant CFTRs may be useful in the characterization of defects in chloride transport as well as the response to chronic CFTR modulator treatments. The strategy of sequential exposure of modulators with acute actions may be extended beyond those utilized in this study by including additional modulators with unique mechanisms of action, and this could provide even more information regarding the details of the regulation of function in normal and mutant CFTRs. While experiments in primary airway epithelial cells should have increased physiological relevance over experiments in FRT cells exogenously expressing CFTRs, both may be important for fully understanding the functional impacts of both CFTR modulators and CFTR mutations. Future studies will involve exploring the sequential application of additional CFTR activators and potentiators during the analysis of primary airway cultures and FRT cells expressing both normal and mutant CFTRs with and without chronic CFTR modulators; these studies will more clearly demonstrate the potential of utilizing this approach for furthering our knowledge regarding CFTR.

## 4. Materials and Methods

### 4.1. Cells and Culture

Primary human nasal epithelial cells (HNECs) were obtained from brushings of the inferior turbinate using an IRB-approved protocol (NGP2021-0023) and expanded using conditional reprogramming culture methods utilizing the ROCK inhibitor Y-27632 (#A3008, Apex BIO, Huston, TX, USA) and irradiated 3T3 fibroblasts, as previously described [29,38,39]. For differentiation at the air–liquid interface (ALI), expanded cells were plated at a density of 250,000 cells/cm^2^ on 6.5 mm permeable Transwell inserts (#3270, Corning Life Sciences, Tewksbury, MA, USA) coated with bovine Type I collagen (#5005-b, Advanced BioMatrix, Carlsbad, CA, USA), grown while submerged for 2 days in PneumaCult™-Ex Plus (#05040, STEMCELL Technologies, Vancouver, BC, Canada), then cultured at ALI using PneumaCult™-ALI media (#05001, STEMCELL Technologies) for 3–5 weeks. For 24 h treatment with forskolin, cells were treated with media containing either DMSO alone (vehicle; #BP231-100, Fisher Chemical, Pittsburgh, PA, USA) or 10 µM forskolin (#11018, Cayman Chemical, Ann Arbor, MI, USA).

Fischer rate thyroid (FRT) cells expressing normal CFTR were a generous gift from Dr. Eric J. Sorscher (Emory University, Atlanta, GA, USA) [40]. FRT cells were plated at a density of 150,000 cells/cm^2^ on 6.5 mm permeable Transwell inserts. FRT cells were cultured in modified Ham’s F-12 media (Coon’s modification; #F6636-1L, Sigma-Aldrich, St. Louis, MO, USA) with 10% fetal bovine serum (#FB12999102, Fisher Chemical, Pittsburgh, PA, USA), 1% penicillin/streptomycin (#30-002-CI, Corning Mediatech, Manassas, VA, USA), and 0.2% hygromycin (#10689010, Invitrogen, Waltham, MA, USA) for 5–7 days after plating and prior to analysis. FRT cells expressing functional CFTR were utilized at passage 12 or 13, and cells transfected with the empty vector were utilized at passage 7.

### 4.2. Electrophysiology

Electrophysiological analyses were performed in an Ussing Chamber (Physiologic Instruments, Reno, NV, USA) under current clamp (0 μA) conditions with intermittent current pulsing (200 ms pulses at ±5 μA). Transepithelial current (∆I_T_), resistance (∆TEER), and potential difference (TEPD) values were continuously monitored. I_T_ values for data obtained in HNECs are shown in all figures, and the TEPD and TEER values for Figure 1, Figure 2 and Figure 3 are shown in Supplemental Figures S1–S3, respectively. Cells analyzed under symmetrical chloride were bathed in a modified Ringer’s solution (120 mM NaCl, 10 mM D-Glucose, 3.3 mM KH_2_PO_4_, 0.83 mM K_2_HPO_4_, 1.2 mM MgCl_2_, 1.2 mM CaCl_2_, 25 mM NaHCO_3_, pH 7.4), maintained at 37 °C, and gassed with 5% CO_2_/95% O_2_. For cells analyzed under a chloride gradient, a solution with gluconate substituted for chloride (115 mM NaC_6_H_11_O_7_, 10 mM D-Glucose, 3.3 mM KH_2_PO_4_, 0.83 mM K_2_HPO_4_, 1.2 mM MgSO_4_, 5 mM CaC_6_H_11_O_7_, 25 mM, and NaHCO_3_, pH 7.4) was applied to the side of the transwell containing the cells (for HNECs, this is defined as the apical surface). During analysis of HNECs, the following concentrations of compounds were utilized as indicated: apical 10 μM amiloride (#J62168, Alfa Aesar, Haverhill, MA, USA); apical 1 μM VX-770 (#S1144, Selleck Chemicals, Huston, TX, USA); apical 10 μM C_act_-A1 (#505985, EMD Millipore, Burlington, MA, USA), 20 μM forskolin (#11018, Cayman Chemical)/100 μM IBMX (#I5879, Sigma-Aldrich) to both chambers; apical 20 μM CFTR(inh)-172 (Cystic Fibrosis Foundation Therapeutics CFTR Compound Program). During the analysis of FRT cells, the same concentrations of drugs were utilized and applied to both chambers.

### 4.3. Data Analysis and Statistics

The results in each experimental HNEC data set depict data generated from cultures from a single donor and are representative of data obtained from multiple unique donors. For drug responses, all changes in values are absolute values to allow for differences amongst groups to be more clearly displayed. All changes in values are calculated as the maximum change in response to the stimuli, with the exception of the “Extended F/I” values shown in Figure 1 and Figure 2.

To test the distribution of data sets for normality, values of changes in current in response to amiloride from technical replicates were analyzed using the Shapiro–Wilk normality test (α = 0.05), with all sets passing this test. For comparisons of groups, t tests were performed, with resulting *p* values less than or equal to 0.05 considered significant. All statistical analyses were performed using Prism 10 (v10.3.1, GraphPad Software, San Diego, CA, USA).

## Data Availability

The original contributions presented in this study are included in the article/supplementary material. Further inquiries can be directed to the corresponding author.

## Supplementary Materials

The following supporting information can be downloaded at: https://www.mdpi.com/article/10.3390/ijms26020471/s1.
