# Supplementary material for: VX-770, Cact-A1, and Increased Intracellular cAMP Have Distinct Acute Impacts upon CFTR Activity
Source: Int J Mol Sci. 2025 Jan 8;26(2):471. doi: 10.3390/ijms26020471 (PMC11764695; doi:10.3390/ijms26020471)
Supplement: Supplementary file 1 [file ijms-26-00471-s001.zip › Supplemental Figure 2_v3.pdf]

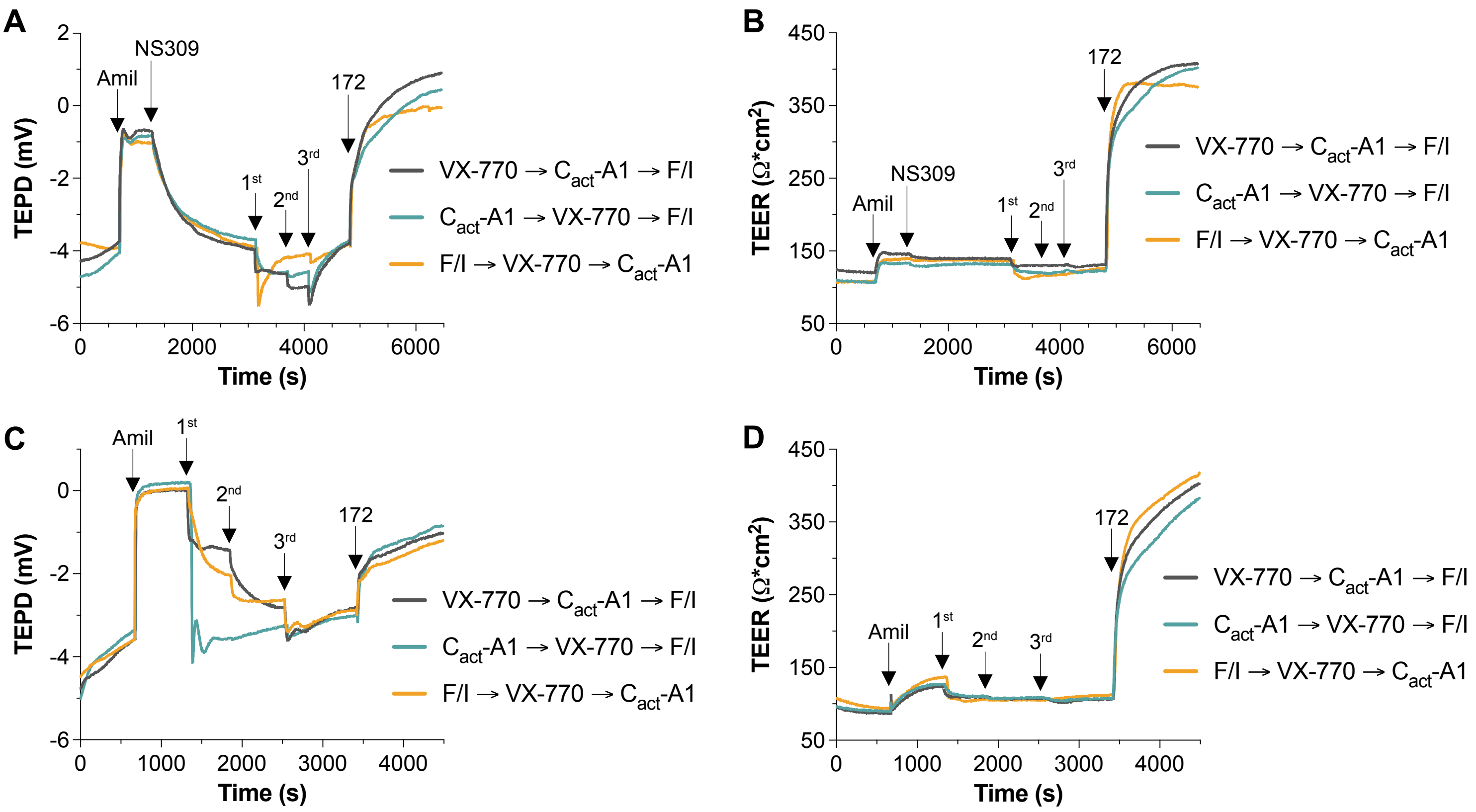


**Supplemental Figure 2.** Transepithelial potential difference (TEPD) (A, C) and transepithelial electrical resistance (TEER) (B, D) traces corresponding to data shown in Figure 2A and 2C.
