# Supplementary figures and images for: VX-770, Cact-A1, and Increased Intracellular cAMP Have Distinct Acute Impacts upon CFTR Activity
Source: Int J Mol Sci. 2025 Jan 8;26(2):471. doi: 10.3390/ijms26020471 (PMC11764695; doi:10.3390/ijms26020471)

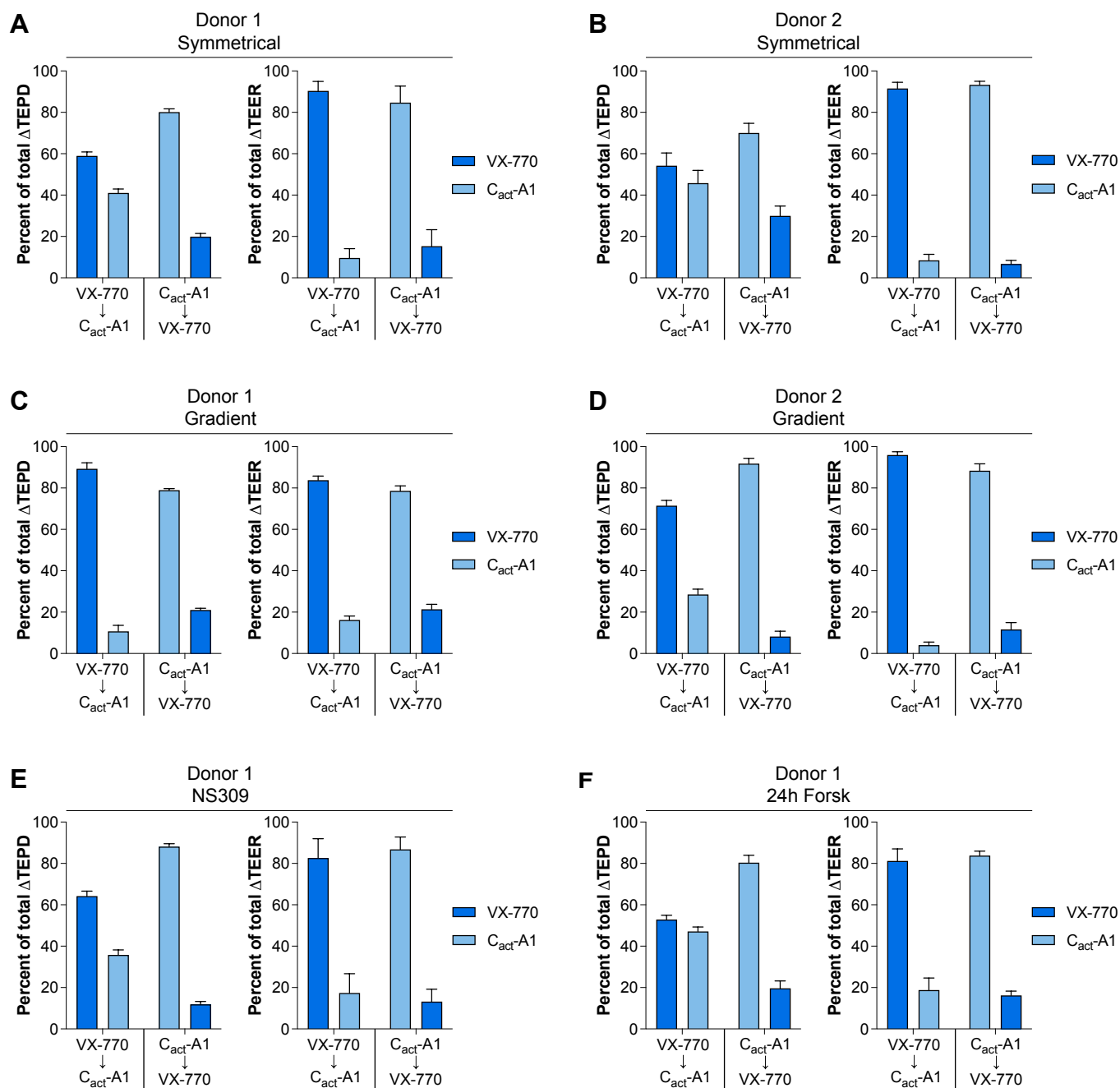

**Supplemental Figure 3.** Changes in TEPD and TEER corresponding to data shown in Figure 3.

Supplement: Supplementary file 1 [file ijms-26-00471-s001.zip › Supplemental Figure 3_v3.pdf]
